# Factors influencing the uptake of short-term contraceptives among women in Afghanistan

**DOI:** 10.1038/s41598-022-10535-y

**Published:** 2022-04-22

**Authors:** Ahmad Siyar Noormal, Volker Winkler, Ali Maisam Eshraqi, Andreas Deckert, Iftekhar Sadaat, Peter Dambach

**Affiliations:** 1 Heidelberg Institute of Global Health, Im Neunheimer Feld 365, 69120 Heidelberg, Germany; 2 Kabul University of Medical Sciences, Kabul, Afghanistan; 3 Ministry of Public Health, Kabul, Afghanistan

**Keywords:** Public health, Epidemiology

## Abstract

The aim of this study is to assess factors that influence the uptake of short-term contraceptives among married women aged between 15 and 49 years in Afghanistan. The cross-sectional Afghanistan 2015 Demographic and Health Survey provided the dataset for this analysis. We included 22,974 women and applied multivariable logistic regression to investigate the influencing factors for the uptake of short-term contraceptives. 92% of Afghan women knew at least one type of short-term contraception but only 17% were using short term contraceptives. Short term contraceptive use was most prevalent among women in the age group between 30 and 40 who were educated, employed, and rich. Most of the users were living in the western parts of Afghanistan and women from the Balooch and Pashtun ethnic groups were most likely to use short-term contraceptives. Media exposure and women empowerment were also positively associated with the use of short-term contraceptives. We did not find an association with living in urban or rural settings. Contraception promotion in Afghanistan requires multisectoral efforts, tailored to the needs of women from low and middle socioeconomic strata. Health promotion activities, empowering women, strengthening education, and training of service providers on effective counseling are options that should be considered to improve the current situation.

## Introduction

According to the World Health Organization, ‘Family Planning (FP) allows people to attain their desired number of children and determine the spacing of pregnancies. It is achieved through the use of contraceptive methods and the treatment of involuntary infertility’. FP is an effective approach to reduce maternal and child mortality^1,2^. Studies have shown that the use of contraceptives has prevented approximately 272,000 maternal deaths worldwide, which is equivalent to a 44% reduction in maternal mortality. Other studies have revealed that contraception use is strongly associated with a reduction in neonatal deaths^2–5^.

Globally, about 12% of married women have an unmet need for contraception with the majority of them living in low and middle income countries^6^. Recent data showed that in 2017 out of 206 million pregnancies in developing countries, around 43% were unintended. According to WHO, about 214 million women of reproductive age who want to avoid pregnancy are not using any method of contraception^7^. This unmet need for contraception results in millions of unwanted pregnancies^7^. A study showed that promotion of contraceptives in developing countries over the past 2 decades has avoided up to 40% of maternal deaths simply by reducing the number of unwanted pregnancies^8^.

Depending on the source of literature, the maternal mortality ratio in Afghanistan lies between 1291^9^ and 638^10^ in year 2017, making them rank among the highest worldwide. Traditionally, Afghans tend to have large families, and even though the fertility rate has declined in recent years from 7.4 in 2000, to 5.1 in 2018^11^, the total fertility rate is still considered high compared to its South Asian neighbors^12^.

Over the past decade, there was an increase in the prevalence of contraceptive use from 7% in 2003 over 11% in 2012 to a still low 23% in 2015^13^. The report of the Afghanistan Demographic and Health Survey (AfDHS) revealed that around 53% of women were not in need of contraception and around 24% had an unmet need for contraception which is considered very high^9^. Given Afghanistan’s high fertility rate and relatively high prevalence of unmet need for contraception, the prevalence of contraception is considered low compared to other countries in the region and all over the world^14^.

Contraceptives may either be classified into modern and traditional methods, or according to the duration of their effectiveness into long term, short term, and permanent contraceptives. According toAfDHS, in 2015, in Afghanistan 95% of married women and 92% of married men know at least one method of FP^9^. Despite this high level of awareness, the prevalence of contraception utilization remained low at 23%, with 16% being short-term contraceptives, 3% being traditional methods, and less than4% being intrauterine devices, sterilization and implants^9^. The report also showed that the met need for contraception was equally distributed for spacing and limiting, each responsible for 11%. However from 24% unmet need, 18% was for spacing and 7% for limiting, which adds more importance to short-term methods in the country.

In Afghanistan, very few studies^15–17^ assessed the predictors of family planning modern methods, but neither particularly explored the determinants of short-term methods, amidst their utmost importance.

In this study we hypothesize that the use of short-term contraceptive methods in Afghanistan is influenced by women’s sociodemographic characteristics, exposure to FP Information, and women’s empowerment. Therefore, we aimed to assess the factors that influences the uptake of short-term contraceptives among married women aged between 15 and 49 years in Afghanistan.

## Methodology

Pills, injectables, and male condoms, which are classified as short-term methods^18^, were by far the most widely known and practiced methods among both women and men, and we therefore focused our analysis only on these short-term methods. To gain a better understanding of the context, our analysis explicitly focuses on predictors of and barriers to the use of short-term contraceptives. We used the nationally representative (except for Baghlan Province) AfDHS dataset. Data collection was carried out between July 15, 2015, and February 23, 2016. The AfDHS 2015 used an updated version of the Household Listing Frame as sampling frame^19^, which included information from 25,974 enumeration areas (EAs). The required number of households was estimated for each EA using location (province, and district) and type of residence (urban or rural)^9^.

The stratified two stage random sample design resulted in representative estimates of demographic and health indicators. Details on the data collection process can be found elsewhere^9^. All ever married women between the age of 15–49 years who were permanent residents of the selected households or visitors who stayed in the households the night before the survey were eligible for the survey. All participants have agreed to take part in the survey and signed the informed consent forms (For minor participants informed consent has been obtained from their legal guardians). Women who were pregnant at the time of survey were excluded for this analysis, because they will not utilize contraception. The data from this survey are openly available under https://dhsprogram.com/data/availabledatasets.cfm.

**Figure 1:**
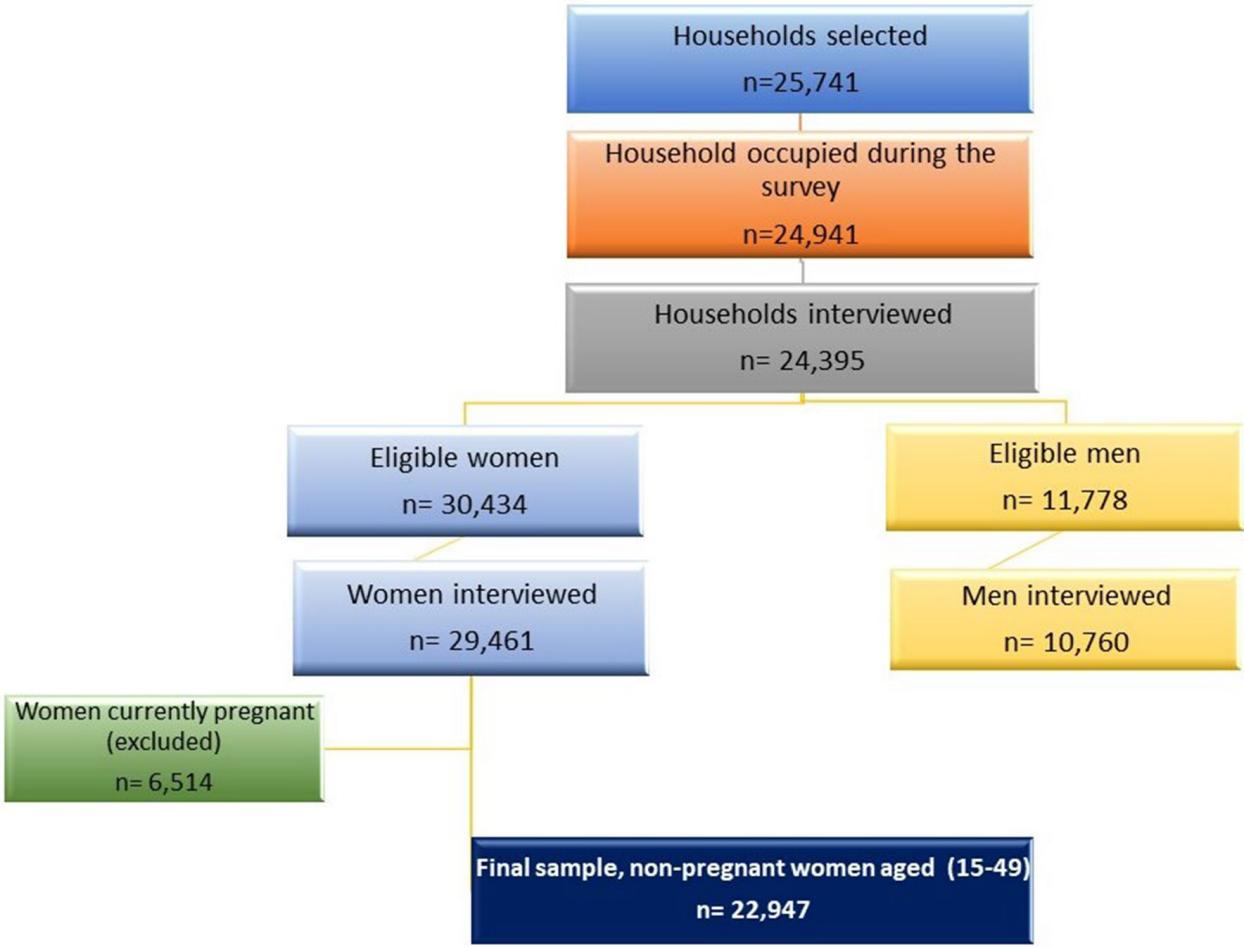
Flowchart of participants.

A total of 29,461 out of 30,434 eligible women (response rate 96.8%) were interviewed in the survey. Out of these, we excluded 6514 women (22.1%) who were pregnant at the time of data collection, hence, remained with a total of 22,947 women (Fig. 1).

The survey protocol was approved by the Inner-City Fund (ICF) Institutional Review Board and the Ministry of Public Health of Afghanistan, and the survey methods were performed in-line with the relevant guidelines and regulations^9^.

The outcome variable for this analysis was the current use of short-term contraceptives as binary indicator. As potential explanatory variables we considered: age, education, wealth index, occupation, place of residence, ethnicity, region, media exposure, and women empowerment. Media exposure was created from the questions that asked the women if they had heard of FP messages from TV, radio, or magazines. Based on this, the participants were categorized as ‘exposed to FP information’ if they affirmed to at least one of these questions. The women empowerment variable was created based on questions about women’s involvement in household decisions regarding income, purchases, family visits, and their health. Women were categorized as ‘partially empowered’ if they were involved in 1 or 2 of the decisions, and ‘fully empowered’ if they were involved in 3 or 4 of the decisions.

For the descriptive analysis we used the Pearson’s Chi square test to assess the existence of an association between the use of short-term contraceptives and the explanatory variables. Then we used logistic regression to estimate odds ratios for the independent variables.

Since the dataset was large, and all the variables were found to be significant at level of 95% confidence interval, we decided for more strict criteria to minimize the risk of chance and set the statistical significance to α = 0.01 and calculated 99% of confidence intervals with robust standard errors. Moreover, all the predictors were significant in the univariate model except place of residence, therefore we performed the full model analysis. We first decided to establish the final model through the backward elimination method, but since the data was hardly changing with that, we included all the variables in the final adjusted model. Stata version 12 IC was used for analyses.

### Ethical approval

Since we used the secondary data from DHS, we did not need ethical clearance for this analysis as this survey was already approved by the IRB of Afghan Ministry of Public Health. Participant have agreed to take part in the survey and signed the consent form.

## Results

### Sociodemographic distribution and use of short-term contraceptives

Table 1 shows that 20.3% (4656 women) of the participants (non-pregnant women) in this survey were between 25 and 29 years old, followed by the age group of 20–24 (18% equal to 4270 women). Over 85% (19,690) of women were illiterate, and around 87% (20,070) of the participants were unemployed. Almost three quarters of the participants belonged to Pashtun and Tajik ethnic groups. Three quarter (74.4% equal to 17,073) of the women were living in rural areas. The number of women who did not have access to FP information was almost twice as high as of those who had access. Moreover, around one third (8023) of the participants were classified as unempowered, 23.5% (5396) as partially empowered, and 37.7% (8,662) as fully empowered.

**Table 1:** Socio-demographic characteristics of women tested against the outcome short-term contraceptive Use.

Variables	Total number of women	Use of short-term contraceptives		Unmet need for contraception		p-value
		Number	Percentage	Spacing	Limiting	
**Age of mother**						< 0.0001
15–19	1189 (5.18%)	84	7.1%	380 (32.87%)	15 (1.30%)	
20–24	4270 (18.6%)	584	13.7%	1413 (33.71%)	90 (2.15%)	
25–29	4656 (20.3%)	881	18.9%	1425 (30.94%)	200 (4.34%)	
30–34	3414 (14.9%)	712	20.9%	808 (24.11%)	283 (8.45%)	
35–39	3495 (15.2%)	739	21.1%	541 (15.85%)	476 (13.95%)	
40–44	2903 (12.7%)	562	19.4%	221 (7.84%)	326 (11.56%)	
45–49	3020 (13.2%)	332	11.0%	86 (2.95%)	211 (7.24%)	
**Education level**						< 0.0001
No Education	19,690 (85.8%)	3200	16.3%	4801 (21.20%)	1439 (7.47%)	
Primary	1519 (6.6%)	322	21.2%	333 (22.32%)	87 (5.83%)	
Secondary	1345 (5.9%)	280	20.8%	362 (27.42%)	59 (4.47%)	
Higher education	393 (1.7%)	92	23.4%	98 (25.32%)	16 (4.13%)	
**Wealth Index**						< 0.0001
Poorest	4342 (18.9%)	646	14.9%	997 (23.58%)	321 (7.59%)	
Poorer	5041 (22.0%)	673	13.4%	1108 (22.47%)	347 (7.04%)	
Middle	4917 (21.4%)	716	14.6%	1070 (22.20%)	332 (6.89%)	
Richer	4974 (21.7%)	912	18.3%	1117 (22.95%)	345 (7.09%)	
Richest	3673 (16.0%)	947	25.8%	582 (16.14%)	256 (7.10%)	
**Women occupation**						< 0.0001
Employed	2858 (12.4%)	400	14.0%	615 (21.98%)	142 (5.08%)	
Unemployed	20,070 (87.5%)	3492	17.4%	4254 (21.67%)	1457 (7.42%)	
Missing	19 (0.1%)					
**Place of residence**						< 0.0001
Rural	17,073 (74.4%)	2582	15.1%	3777 (22.62%)	1090 (6.53%)	
Urban	5874 (25.6%)	1312	22.3%	1097 (19.06%)	511 (8.88%)	
**Ethnicity**						< 0.0001
Pashtun	9531 (41.5%)	1656	17.4%	2045 (21.90%)	518 (5.55%)	
Tajik	7141 (31.1%)	1432	20.1%	1364 (19.53%)	615 (8.81%)	
Hazara	2258 (9.8%)	428	19.0%	442 (20.13%)	187 (8.52%)	
Uzbek	1604 (7.0%)	175	10.9%	384 (24.58%)	143 (9.15%)	
Turkmen	474 (2.1%)	33	7.0%	153 (32.83%)	38 (8.15%)	
Nooristani	877 (3.8%)	9	1.0%	266 (30.50%)	7 (0.80%)	
Baloch	291 (1.3%)	74	25.4%	39 (14.03%)	23 (8.27%)	
Pashai	405 (1.8%)	28	6.9%	110 (27.57%)	36 (9.02%)	
Other	325 (1.4%)	56	17.2%	60 (18.93%)	29 (9.15%)	
Missing	41 (0.2%)	1656				
**Region**						< 0.0001
Central	3991 (17.4%)	806	20.2%	629 (16.12%)	372 (9.53%)	
North Eastern	2562 (11.2%)	296	11.6%	660 (26,62%)	242 (9.76%)	
North Western	3157 (13.8%)	330	10.5%	721 (23.31%)	280 (9.05%)	
Eastern	2980 (13.0%)	239	8.0%	727 (24.66%)	164 (5.56%)	
Western	3439 (15.0%)	1040	30.2%	510 (15.16%)	198 (5.89%)	
South Eastern	3531 (15.4%)	424	12.0%	911 (26.37%)	137 (3.97%)	
South Western	3287 (14.3%)	759	23.1%	716 (22.31%)	208 (6.48%)	
**Access to family planning information**						< 0.0001
Yes	8451 (36.8%)	1895	22.4%	1639 (19.72%)	660 (7.94%)	
No	14,411 (62.8%)	1989	13.8%	3217 (22.86%)	936 (6.65%)	
Missing	85 (0.4%)					
**Women empowerment**						< 0.0001
Not empowered	8023 (35.0%)	1298	14.60%	6213 (71.16%)	525 (6.01%)	
Partially empowered	5396 (23.52%)	1159	21.48%	1039 (19.68%)	405 (7.67%)	
Fully empowered	8662 (37.75%)	1.437	16.59%	1842 (21.82%)	671 (7.95%)	
Missing	866 (3.8%)					
Total	22,947 (100%)	3894	16.96%			

Overall use of short-term contraceptives was quite low: among the total of 22,947 participants, 17% used short term contraceptives and 6% employed long-acting methods and traditional methods.

Table 1 shows that the proportion of unmet need for spacing is higher than for limiting in all categories. Considering the age groups, the unmet need for spacing was highest (33.71% equal to 1413 women) in the age group of 20–24, and lowest (2.95% equal to 86 women) in the 45–49 age group, while for limiting this trend showed different pattern with the lowest (1.30% equal to 15 women) in the 15–19 age group and highest (13.95% equal to 476 women) in the age group of 35–39.

Distribution of unmet need by ethnicity and regions was also heterogeneous. Turkmen women had the highest (32.83%, or 153 women) unmet need for spacing while Balooch women had the lowest (14.03%, or 39 women). Similarly the unmet need for limiting varied from 9.15% (143 person) among Uzbek women to 0.8% (7 person) in Nooristani women.

Women living in the northeastern region had the highest (26.62%, or 660 women) unmet need for spacing, while those in the western region had the lowest (15.16% equal to 510 women).

Women empowerment showed remarkably different prevalences of unmet need. Unempowered women had the highest (71.16% equal to 6213 women) unmet need for spacing, while partially and fully empowered women had 19.68% (1039 women) and 21.82% (1842 women) respectively.

The unmet need was almost equally distributed among women with varying levels of education, employment status, wealth status, living settings, and access to FP information.

### Multivariable logistic regression analysis

The result from the multiple logistic regression revealed an association between all the predicting factors and use of short-term contraception. The only factor which was not significantly (p value = 0.45) associated was the place of residence and we did not find any significant difference in the use of short-term contraception among women living in urban and rural settings (Table 2).

**Table 2:** Crude and adjusted odds ratios and the corresponding confidence intervals of contraceptive use regressed on the predictor variables (link function: logit).

Variables	Bivariate Model		Full Model		
	Crude Odds ratio	99% Confidence Interval (CI)	Adjusted Odds ratio	99% Confidence Interval (CI)	p-value
**Women age group**					< 0.001
15–19	0.62	0.48–0.79	0.51	0.36–0.71	
20–24	1.28	1.11–1.48	1.18	0.97–1.44	
25–29	1.89	1.65–2.16	1.90	1.58–2.29	
30–34	2.13	1.85–2.46	2.14	1.77–2.60	
35–39	2.17	1.89–2.50	2.12	1.75–2.56	
40–44	1.94	1.68–2.25	1.94	1.59–2.37	
45–49	(1) Reference		(1) Reference		
**Education level**					0.002
No education	(1) Reference		(1) Reference		
Primary	1.39	1.29–1.58	1.27	1.05–1.52	
Secondary	1.35	1.18–1.55	1.21	0.99–1.48	
High school	1.58	1.24–1.99	1.06	0.74–1.52	
**Ethnicity**					< 0.001
Pashtun	(1) Reference		(1) Reference		
Tajik	1.19	1.10–1.29	0.94	0.83–1.08	
Hazara	1.11	0.99–1.25	0.94	0.79–1.12	
Uzbek	0.58	0.49–0.69	0.87	0.67–1.15	
Turkmen	0.36	0.25–0.51	0.55	0.33–0.94	
Nooristani	0.05	0.02–0.09	0.10	0.04–0.24	
Balooch	1.62	1.24–2.12	1.23	0.84–1.81	
Pashai	0.35	0.24–0.52	0.52	0.30–0.88	
Other	0.99	0.74–1.33	1.02	0.67–1.56	
**Wealth Index**					< 0.001
Poorest	(1) Reference		(1) Reference		
Poorer	0.88	0.78–0.99	1.11	0.94–1.31	
Middle	0.98	0.87–1.09	1.29	1.08–1.53	
Richer	1.28	1.15–1.43	1.56	1.31–1.86	
Richest	1.99	1.78–2.22	1.99	1.59–2.46	
**Women occupation**					< 0.001
Unemployed	(1) Reference		(1) Reference		
Employed	0.77	0.69–0.86	1.20	1.01–1.42	
**Place of residence**					0.4519
Urban	(1) Reference		(1) Reference		
Rural	0.62	0.57–0.67	1.04	0.89–1.21	
**Region**					< 0.001
Central	(1) Reference		(1) Reference		
Northeastern	0.52	0.45–0.59	0.56	0.46–0.69	
Northwestern	0.46	0.40–0.53	0.50	0.40–0.62	
Eastern	0.34	0.29–0.40	0.49	0.39–0.62	
Western	1.71	1.54–1.90	2.06	1.76–2.41	
Southeastern	0.54	0.47–0.61	0.54	0.45–0.65	
Southwestern	1.19	1.06–1.33	1.30	1.09–1.55	
**Media exposure**					< 0.001
Do not have access to FP information	(1) Reference		(1) Reference		
Have access to FP information	1.81	1.68–1.94	1.43	1.29–1.59	
**Women empowerment**					< 0.001
Not empowered	(1) Reference		(1) Reference		
Partially empowered			1.55	1.36–1.75	
Fully empowered			1.23	1.09–1.38	

As shown in Table 2, the likelihood of using contraceptives was lowest in the youngest age group, compared to the oldest, and increased with age up to 30–39 years, while it decreased again afterwards. Interestingly women in the age group of 30–40 were nearly 4 times more likely to use short-term contraceptives compared to younger women and 2 times more than those at older ages.

Education level was also significantly associated with the use of short-term contraceptives. Surprisingly women with primary education were more likely to adhere to the use of contraceptives (AOR 1.27 CI 1.05–1.52) compared to illiterate women, however there was no significant difference in the uptake of short-term contraception between those who had secondary and higher education and those with no education. An increase in the wealth index was also significantly associated with the uptake of short-term contraceptive use. The richest women were 2 times (AOR 1.99, CI 1.59–2.46) more likely to use short-term contraception compared to the poorest. Employed women were more likely (AOR 1.2, CI 1.01–1.42) to use short-term contraceptives compared to unemployed women. Region of residence equally played an important role in the uptake of contraceptives in Afghanistan (Table 2). The usage of short-term contraceptives was very high among women in the western region (AOR 2.06, CI 1.76–2.41) compared to the other 6 regions. Women living in the Northeastern, Northwestern, Eastern, and Southeastern regions were least likely to use short term contraceptives compared to those living in the central region. Regarding ethnicity, we considered the Pashtun ethnic group as a reference for having the largest proportion in the dataset and compared the other ethnic groups with this category. Only Balooch women did use short-term contraceptives significantly more often (AOR 1.23, CI 0.84–1.81) compared to the reference ethnic group, while Nooristani and Pashai women had the lowest ratios compared to the Pashtun group. Access to FP information also showed a strong association with short term contraceptive uptake. Women who had access to FP information were 1.4 (AOR 1.43, CI 1.29–1.59) times more likely using short-term contraceptives compared to those with no access. We also found an association between women empowerment and uptake of short-term contraception as partially empowered women were 1.55 (AOR 1.55, CI 1.36–1.75) times and fully empowered women were 1.23 (AOR 1.23, 1.09–1.38) times more likely to use short-term contraception compared to unempowered women. Interestingly we found that partially empowered women were more likely using short-term contraception than fully empowered women.

## Discussion

Our results highlight that the majority of the women’s sociodemographic characteristics (age, literacy, marital status, wealth, ethnicity, region, access to FP information and women empowerment) were significantly associated with short-term contraceptive use, which supports our hypothesis. These results mostly follow the findings of several studies in the region and all over the world^20–24^, however to our surprise the place of residence did not make a significant difference which was not consistent with what was already known from the region^17,25^. Interestingly the association with living setting disappeared after adjusting for other characteristics, especially wealth. There could be many contextual implications for this result such as social and religious norms, financial dependency of women, cultural restrictions, and stigmatization towards seeking FP services, all of which affect the uptake of contraception in the entire population independent of their living setting. Moreover, short-term methods are easy to access (could be found easily in dispensaries), and easy to apply (they do not require skilled healthcare workers), therefore those living in rural areas are more likely to adhere to short-term methods, thus we did not find any significant difference in the uptake of short-term contraception between these two groups.

Similar to several studies in the region and other developing countries^21,22,26^, our study revealed a significant association between age and contraceptive use. In our study, the prevalence of short-term contraceptive uptake was higher among women aged between 30 and 40 years old while women younger than 20 years had the lowest rate. This could be due to several reasons: they are newly married and do not want to avoid pregnancy; these women are less aware of the methods^27^; reproductive health is still a taboo^28^ and thus, young women feel ashamed to discuss such issues and to seek FP services.

Region and ethnicity also play a role in the usage of contraception. There was a remarkable difference in contraception usage across different regions, as women living in the western parts of the country were 4 times more likely to use contraceptives compared to those who were living in the eastern region. Surprisingly a huge difference was found in the uptake of contraception among ethnic groups with Balooch women using contraception nearly 13 times more than Nooristani women. The basic conception behind these variations could be the geography and security concerns which may limit the provision of health services in these regions. Since Nooristan is a deprived and mountainous province in the east of Afghanistan, and it was highly insecure in 2015, this might explain the low uptake. The Balooch on the other had, live near the border between Afghanistan and Iran in the west of the country where there is better access to health services which may a strong contributor to the observed high contraceptive uptake.

Access to FP information is an important determinant of contraceptive uptake. In the DHS survey women were already considered as knowledgeable if they were able to name at least one method of FP. Having stated in the DHS survey to know about contraceptives does not necessarily imply knowledge about its correct application, nor does it determine actual use. What we looked at in our study was their exposure to FP information which we assumed, increased their knowledge about the methods, the benefits, side effects and further aspects of FP. This finding is consistent with other studies conducted in the region and other developing countries^29–31^. Moreover if we look towards other aspects, there could be discrepancies between knowledge and empowerment. The strong cultural anchor of having big families plays an important role which puts women under pressure to bear as many children as possible and not use contraceptives, even if they have knowledge on contraceptives. In our study we only looked at media exposure, and due to a huge number of missing values in the dataset, we did not include other channels (exposure at the health facility and information through community health workers). We found a significant association between media exposure and short-term contraceptive uptake, and we believe that if we had been able to include other channels into our analysis, the association might have become stronger.

Women empowerment has already been widely known as an important indicator which contributes to many health outcomes and similarly, we found a strong association between empowerment and use of short-term contraception. Gender equality provides the opportunity to a woman to freely discuss and decide about major health aspects specifically the reproductive health issues. Moreover, those women who have autonomy in their households are in a better position to have access to health services. An Interesting finding in our study was that partially empowered women were more likely using short-term contraception compared to fully empowered and unempowered. This statement can be supported by a culturally bonded norm where in Afghan families, women are getting more empowered and autonomous as they get grow older and older. Thus, we can assume that those women who are empowered are more of old ages of older ages, therefore and thus they are more likely to use contraception as we already also discussed it in the age category, however the relatively lower odds of contraception uptake in fully empowered women can be justified by their very old ages in which they might prefer to use long-term or permanent methods more often frequently than short -term ones.

### Barriers of contraception uptake

Poor access to contraception, insufficient information about free provision of FP services, fear of side effects, insecurity, cultural and religious disagreements, and gender issues are considered potential barriers for contraception uptake in Afghanistan^16,28,32^. Since the majority of the population in Afghanistan is living under the poverty line^33^, we would conclude poor economic status as a barrier to contraception uptake. Furthermore, cultural beliefs such as preference of male children in certain regions of Afghanistan, social stigma and taboos against contraception, early marriage, familial pressures, and religious beliefs that stopping pregnancy is forbidden in Islam^28^, are important barriers for contraception uptake. Additionally, some people believe that large families are desirable for some reasons such as having someone to help parents in their old ages, securing economic status by having more people to work, and compensating possible losses due to conflicts.

Since we only conducted a quantitative analysis, we identified some of the potential barriers, but there might be some underpinning factors and barriers which need investigation using qualitative research methods.

### Recommendations

FP is a wide area which requires a multisectoral collaboration and approach to promote in the society. Considering the country’s current emergency, particularly the suspension of world bank donation to the health system, there is a crucial need for efforts to strengthen the entire health system. As a result of the crisis, it is predicted that around 2331 health centers will be closed, and approximately 25,000 health workers including 7500 female workers will be unemployed in near future^34^. Moreover, with the decision of the new government, female workers got unemployed and banned from education. This lack of women’s access to health services, unavailability of essential medicines, lack of female health staff, and women unemployment will affect the entire health system specifically the health of mothers and children, contributing to an increment in the maternal mortality ratio as well as child and neonatal deaths. Therefore, advocacy for donors to support the area of mother and child health is the first suggestion for ensuring access and availability of health services, including FP methods. Community based provisions and the use of community health workers (CHWs) to educate both men and women and provide FP services is recommended. Mass media should be used to promote and advocate for FP. Since Afghanistan is a country in which religion plays an important role in daily life, one of the effective approaches to advocate for the promotion of FP would be to use religious leaders in the society. Some small-scale initiatives have already been launched in this regard, showing promising results^35^. Inclusion of FP methods as clinical attachments or as part of the medical and nursing curriculum which is currently lacking, as well as regular trainings for service providers on correct and comprehensive counseling are also known to be effective approaches for contraceptive promotion. Improving logistics and supply chain management specifically in the eastern parts of the country would be a crucial step.

There have been many basic improvements in FP in recent decades in Afghanistan, such as the development of the Reproductive, Maternal, Newborn, Child, and Adolescent Health (RMNCH) strategy, a costed implementation plan^36^, and commitment to the vision of FP2020 by increasing the number of health centers providing family planning services, and adding implants to the essential medicine list^37^, we still need to strengthen the initiatives.

We also suggest conducting qualitative research to find out the reason behind non-use and discontinuation of contraceptive methods. Conducting Focus Group Discussions and In-depth interviews not only with women but also with husbands and in-laws about different aspects of FP such as expectations of the society, decision making regarding FP, and level of knowledge on contraception, is highly recommended.

The aforementioned recommendations could serve the policy makers in the future government of Afghanistan to develop and implement interventions based on empirical evidence which may contribute to the improvement of the health sector, especially the field of mother and child health.

### Strengths and limitations

Using DHS data provides us with a trustworthy source and good data quality compared to many single studies. However, in the original study it was assumed that security issues might not have allowed to cover all the selected areas, therefore 101 reserve clusters were selected for replacement and later 70 clusters replaced the areas which were identified insecure. At the end the AfDHS was unable to collect data from Zabul province in the southeastern region due to security concerns. This issue might have resulted in underestimating the national prevalence rate, because Zabul province is mostly occupied by Pashtun women. The large sample size of 22,974 participants provides a solid basis for statistically robust uni- and multivariable analyses, which is corroborated by the strict confidence interval of 99% employed in this study.

On the other hand, the cross-sectional design of the study restricts the establishment of causal relationships between outcome and exposure. Moreover, some of the potential influencing factors such as number of children, distance from the health facility, and number of ANC visits were not included in this study which might have introduced some residual confounding to this analysis. In addition, men were not included in this analysis. Since this dataset only included married women, we can consider this as a potential limitation of the analysis because there might be higher unmet need for contraception among sexually active unmarried women.

Additionally, the dataset had 44 (0.2%) missing values for ethnicity, 19 (0.1%) missing values for occupation, access to FP information had 85 (0.4%) missing values and the women empowerment variable had 866 (3.8%) missing values. Regarding the women empowerment variable, most probably the questions were too sensitive to be answered by all which resulted in information bias.

## Conclusion

Our study revealed a good awareness of the Afghan women on contraceptive methods; however, the use of short-term contraceptives remains low, with only one fifth of the sample population employing them. Low utilization of FP is driven by security, cultural belief, geography, wealth, knowledge, and age. Therefore, policy makers should collaboratively put in place measures to secure funding for the area of mother and child health, enhance mass education on contraception, reduce the barriers on the access to FP services, promote education and employment for women, and deploy efforts to address the misconceptions on FP methods.

## Data Availability

We used the Afghanistan DHS 2015 dataset which is openly available under https://dhsprogram.com/data/available-datasets.cfm.

## Abbreviations

FP: Family Planning
DHS: Demographic and Health Survey
FGD: Focus Group Discussion
WHO: World Health Organization
ICF: Inner city Fund
CHW: Community Health Workers
RMNCH: Reproductive, maternal, newborn, child, and adolescent health
